# Effects of Online Bodyweight High-Intensity Interval Training Intervention and Health Education on the Mental Health and Cognition of Sedentary Young Females

**DOI:** 10.3390/ijerph18010302

**Published:** 2021-01-03

**Authors:** Yao Zhang, Beier Zhang, Liaoyan Gan, Limei Ke, Yingyao Fu, Qian Di, Xindong Ma

**Affiliations:** 1 Division of Sports Science & Physical Education, Tsinghua University, Beijing 100084, China; yao-zhan19@mails.tsinghua.edu.cn (Y.Z.); zbe19@mails.tsinghua.edu.cn (B.Z.); fyy19@mails.tsinghua.edu.cn (Y.F.); 2 Faculty of Kinesiology, Sport, and Recreation, University of Alberta, Edmonton, AB T6G 2H9, Canada; gan@ualberta.ca; 3 School of Medicine, Tsinghua University, Beijing 100084, China; klm20@mails.tsinghua.edu.cn; 4 Vanke School of Public Health, Tsinghua University, Beijing 100084, China; qiandi@tsinghua.edu.cn

**Keywords:** online physical activity intervention, HIIT, health education, mental health, cognitive function, sedentary young females

## Abstract

This study aimed to assess the effectiveness of an online high-intensity interval training (HIIT) intervention and health education on the behaviors, mental health, and cognitive function of sedentary young females. A single-blinded, six-week, randomized controlled pilot trial involving 70 sedentary young Chinese females, aged 18–30 years, was conducted. An intervention group (IG) (*n* = 33) underwent a HIIT intervention and health education, while a waitlist group (WG) (*n* = 37) only received health education. In pre-, mid-, and post-tests, both groups filled out questionnaires about physical activity, sedentary behavior, and mental health. Cognitive functions were assessed at the pre- and post-tests by computer-administered cognitive tests. A mixed-effect model with repeated measures was used to analyze outcomes of interest. The retention rate of the IG and WG was 100% and 78.38%, respectively. The IG were found to have significantly increased rates of moderate-to-vigorous physical activity (MVPA) (M_diff_ = 940.61, *p* < 0.001, 95% confidence interval (95% CI): 576.67, 1304.55) from pre-test to post-test, while the WG demonstrated a more marked reduction in sedentary time (M_diff_ = −73.02, *p* = 0.038, 95% CI: −141.90, −4.14) compared with the IG in the post-test. Moreover, anxiety and stress levels were shown to significantly reduce in the IG over the six-week period (M_diff_ = −4.73, *p* = 0.002, 95% CI: −7.30, −2.15 and M_diff_ = −5.09, *p* = 0.001, 95% CI: −8.29, −1.89, respectively). In addition, we observed a significant improvement in verbal ability (*p* = 0.008, $\eta_{p}^{2}$ = 0.19) following the HIIT intervention and effects of the interaction with time on processing speed (*p* = 0.050, $\eta_{p}^{2}$ = 0.10) and episodic memory (*p* = 0.048, $\eta_{p}^{2}$ = 0.11). Moreover, the IG had better global cognitive performance than the WG in the post-test (M_diff_ = 8.28, *p* = 0.003, 95% CI: 3.06, 13.50). In summary, both an online bodyweight HIIT intervention combined with health education, or health education alone, can effectively improve health-related behaviors, but the behavioral consequences may differ based on the emphasis of different intervention modalities. Furthermore, the “bodyweight HIIT plus health education” modality might be a more promising online intervention strategy to mitigate against negative emotions and improve cognitive function.

## 1. Introduction

Physical activity (PA) is defined as any body movement generated by the contraction of skeletal muscle that raises energy expenditure above the resting metabolic rate [1,2]. It is well acknowledged that physical activity can prevent chronic diseases and improve physical health [3,4]. In addition, converging epidemiological evidence demonstrates that physical activity can prevent psychological disorders and cognitive decline [4,5,6], probably via mechanisms that enhance physical function [4], increase cortical plasticity [7], improve cerebral blood flow [8], and facilitate the release of brain-derived neurotrophic factor (BDNF) [9]. Physical inactivity is defined as non-achievement of the PA guidelines, and any waking behaviors characterized by an energy expenditure ≤1.5 METs (e.g., sitting, reclining, or lying posture) are classified as sedentary behaviors [1,10]. Physical inactivity, which features sedentary behaviors, imposes health risks and results in adverse health outcomes, including declines in mental health status and cognitive function [11,12]. Thus, physical inactivity and sedentary behaviors have become risk factors and have imposed a noticeable public health burden [13].

Physical inactivity is more prevalent in women [14]. In 2016, the global prevalence of insufficient PA in women was 8% higher than that in men [15]. Young women are at a particularly high risk of being sedentary and physically inactive, which can increase stress and anxious emotions, diminish cognitive function, and even elevate all-cause mortality [16,17]. Worryingly, the prevalence of physical inactivity might continue as women age, as the upward tendency of physical inactivity is conspicuous among elder women [18]. Physical exercise is defined as a subcategory of physical activity. It is planned, structured, repetitive activity that promotes physical fitness maintenance or development, and chronic physical exercise can be seen as physical training [1,19]. It is of great health importance to develop appropriate physical training intervention strategies to encourage sedentary young females to start, maintain, and enhance their physical activity behaviors.

Digital media, such as mobile fitness apps and health-related websites, can offer new physical activity intervention strategies for sedentary young females. Online physical training interventions are promising as they involve relatively less contact and lower costs but allow greater flexibility, convenience, and accessibility [20]. Participants may undergo exercise without being limited by location, space, time, or weather, e.g., in a 20-min work break, a sweat-inducing exercise regime can be conducted in a two square meter area by following an online physical training course. Moreover, exercise feelings and obstacles encountered can be shared with other participants from all over the world. As a result, this physical training intervention modality may be especially practical and accessible for sedentary young females living in middle- and low-income nations, and it may help to reduce health inequities. Two recent medium- and long-term randomized controlled trials have shown that online interventions can be effective for improving physical activity behaviors for women [21,22]. However, to the best of our knowledge, no randomized controlled trial has been conducted to investigate the effect of an online physical training intervention on mental health and cognitive function. Furthermore, there is a lack of knowledge on the comparative effects of different intervention modalities, such as high intensity interval training (HIIT) and health education, in the available literature.

Intervention intensity is an important factor in the associations among physical activity, mental health, and cognitive function [23]. It is well-acknowledged that HIIT is a considerably time-efficient and safe exercise modality [24], and bodyweight HIIT is even more space-saving, because it allows participants to undergo exercise of a moderate to vigorous intensity in any place without any equipment [25,26]. Meanwhile, HIIT seems to have better effects on cardiovascular, mental, and cognitive health than other training modalities [26,27,28]. However, very few studies have been conducted to test the effectiveness of short-term bodyweight HIIT combined with health education compared with health education alone on multidimensional health outcomes.

Therefore, we launched a randomized trial to test the effects of online “HIIT + health education” and “health education only” interventions on health-related behaviors, mental health, and cognitive function in a group of sedentary young females. Specifically, in order to determine whether different interventions could contribute to different health outcomes, we focused on increasing moderate-to-vigorous physical activity (MVPA) and reducing sedentary behavior. Hence, this study (1) assessed whether online short-term interventions increase physical activity, decrease sedentary behavior, alleviate negative emotions, and improve primary cognitive function in young Chinese females with self-reported sedentary lifestyles suffering from stress or anxiety; and (2) explored whether two different intervention modalities (i.e., bodyweight HIIT combined with health education vs. health education only with greater concentration on sedentariness reduction) would have dissimilar effects on behavioral, psychological, and cognitive health outcomes. We hypothesized that the two intervention modalities would both improve health-related behaviors but that behavioral changes could differ. It was thought that the “bodyweight HIIT plus health education” intervention would be more effective for improving MVPA while the “health education only” intervention would have a more marked effect on lessening sedentariness. However, HIIT combined with health education modality is inferred to be a relatively better strategy to improve mental health and cognition. This study may provide a theoretical basis for the health benefits of online physical training interventions and broaden the health intervention accessibility for people in need.

## 2. Materials and Methods

### 2.1. Designs

**Figure 1:**
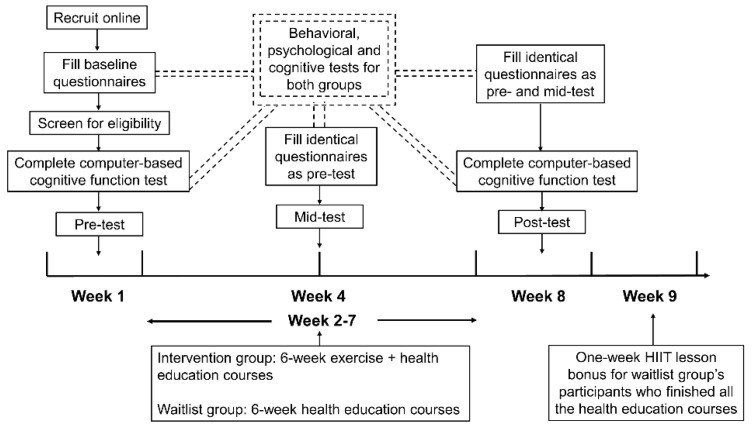
Time axis diagram describing the process of the project.

This study was a single-blinded, online-based, multifactorial physical training intervention, conducted from 29 June 2020 to 31 August 2020, with measurements at three timepoints. Each participant was randomly assigned to either the intervention group (IG) or the waitlist group (WG) using a randomization computer process written by the “sampling” package of R software 3.6.3. Except for the experimenters, no one knew which group each participant belonged to. Given the purposes of our study, participants in the intervention group underwent an intervention involving 6 weeks of HIIT combined with health education, while participants in the waitlist group only received a similar 6-week health education intervention, but the risk of sedentary behavior was given greater emphasis (Details of the 6-week intervention schedules can be seen in Table S1). The outcomes of this study consisted of behavioral and psychological variables measured by structured questionnaires as well as cognitive variables tested by a computer-administered primary cognitive function system. All measurements were done remotely online. This study lasted for nine weeks, during which the active intervention and comparison period was six weeks, while the enrollment, screening, pre-, mid-, and post-tests and additional HIIT lesson bonus took up the remaining three weeks. The details can be seen in Figure 1.

### 2.2. Participants

**Figure 2:**
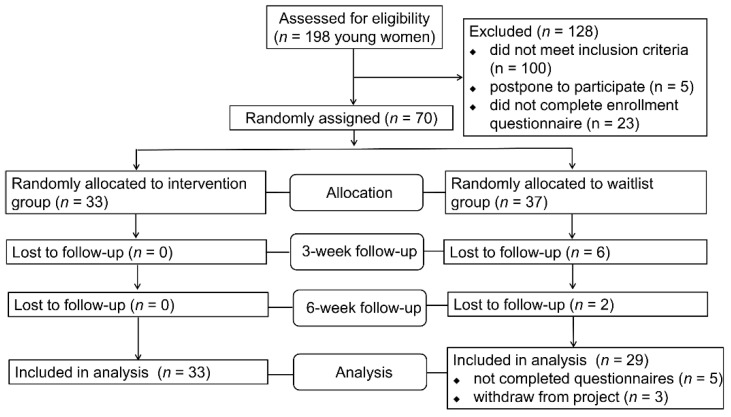
Flowchart showing the study design.

In total, 198 females responded voluntarily to participate in this health-related follow-up project after seeing our recruitment notice online. Demographic characteristics were collected at baseline, including age, weight, height, education level, region, medical history, lifestyle habits, and recent mental status. The inclusion criteria were (a) aged between 18 and 30 years, (b) able to read and write in Chinese, (c) willing and able to perform moderate-to-vigorous physical activity (MVPA), (d) without self-reported cancer diagnosis or history of psychosis, hypertension, or heart disease that would affect their involvement in PA, (e) without regular exercise habits and failure to meet the current international physical activity recommendation (i.e., at least 30 min of MVPA for at least 5 days per week) in the past year, (f) self-reported characteristics of sedentary and anxious/stressed, and (g) intention to improve their quality of life by gaining health-related knowledge or increasing their MVPA. We estimated the minimum sample size required using the “pwr” package of R software 3.6.3, considering an effect size of 0.5, a two-tailed significance level of 0.05, and the typically used power value of 80%. The minimum sample size was determined to be 62 individuals. After screening, a total of 70 participants were eligible for the intervention project. Considering the difficulty with online physical training and our staff resources, we randomly selected 33 participants for the intervention group and 37 for the waitlist group. To ensure effective training and guidance, 33 participants in the intervention group were then divided into three small groups to complete the exercise courses. Finally, in the waitlist group, 3 subjects dropped out and 5 subjects did not fill in the mid- or post-test questionnaires completely. Hence, the final sample size included in the analysis was sixty-two (33 for IG and 29 for WG). Dropout rates for the intervention and waitlist groups were 0% and 21.62%, respectively. More details can be seen in Figure 2.

### 2.3. Interventions

All exercise training and health education courses were conducted by three professional coaches, in the form of Tencent Conference, a Zoom like video conference app, for both groups. The intervention group was received a 6-week home-based intervention, including 2 sessions of bodyweight HIIT-centered exercises and 1 session of health education per week. Participants completed HIIT classes in three small exercise groups on Monday and Thursday, Tuesday, and Friday, and Wednesday and Saturday, respectively. Each exercise session lasted for one hour, from 7 to 8 p.m., including a 5-min warm up, 35 min of HIIT, 10 min of stretching and relaxation exercises, and 10 min of communication. For HIIT, participants performed 30 s exercise repetitions with 30 s of passive recovery between them. The 30-min HIIT exercise sessions were divided into two identical 15-min circuits with 5 min of passive recovery between them. HIIT courses were carried out in the form of body weight training, including core training (e.g., plank), cardiopulmonary function training (e.g., jumping jacks), muscular endurance training (e.g., squats), and so on. More details of the 6-week training plans can be found in Supplementary Table S1.

Referring to previous studies, the interval exercise part of HIIT is typically performed above 80% of peak heart rate (HR_peak_), reaching a high intensity, and there are several methods that can be used for exercise intensity estimation [29,30]. However, since the intervention was online-based, it was hard to monitor real-time heart rate objectively. Instead, a subjective method to guide exercise intensity, the rating of perceived exertion (RPE), was used [29]. Participants in the HIIT classes estimated the intensity of their exercise sessions using a 0–10 RPE Borg Scale [31] which had previously been confirmed to be valid and reliable for monitoring training load [32]. Participants reported their RPE scores at the halfway point and end of each 15-min session. This subjective monitoring method ensured that the exercise intensity level was within the vigorous but safe range (a score of 6–8 on the 0–10 RPE Borg Scale is associated with heavy to very heavy perceived exertion) [31]. Furthermore, interactive communication was conducted in the last 10 min of each HIIT class, where our professional coaches provided real-time corrections according to participant feedback and encouraged them to share their feelings about exercise with each other.

In addition to the physical training intervention, participants in the intervention group received six online health education courses at 11–12 a.m. every Sunday. These included topics on body self-cognition (Week 1); exercise, sedentariness and health (Week 2); nutrition and diet (Week 3); fat-reducing, muscle-gaining, and shaping (Week 4); stretching, meditation, relaxation, and rehabilitation (Week 5); and emotion regulation (Week 6). The analysis only included participants who completed at least 10 exercise sessions and 5 sessions on health education. More details of the 6-week health education plans can be found in Table S1.

The waitlist group only received similar 6-week online health education courses, held at 10–11 a.m. every Sunday. It is worth mentioning that in addition to receiving the same lecture content as the intervention group, the risk of sedentary behavior was emphasized at every class. The analysis only included participants who attended at least 5 of the 6 online health education courses.

### 2.4. Measurements

The main outcomes of this study were five primary cognitive abilities and global cognitive function. The secondary outcomes were behavioral and mental variables, including MVPA, sedentary time, perceived stress, and so on. We used online structural questionnaires to obtain information on demographics, physical activity, sedentary behavior, anxiety and stress in the pre-, mid- and post-tests. Physical activity and sedentary behavior were measured by the short version of the International Physical Activity Questionnaire, which is widely used in different countries for people aged between 15 and 69 years [33,34,35,36]. Participants were asked to classify their typical weekly PA during the previous month into three intensity categories: light (e.g., walking), moderate (e.g., jogging), and vigorous (e.g., lifting and fast cycling) by reporting the number of days per week and duration spent at different PA intensities, as well as the average time spent undergoing sedentary activities on weekdays and weekends. The method for calculating the weekly PA consumption and average amount of sedentary time per day can be seen in supplementary file for measures 2.4.1. Anxiety was measured by the short version of the 10-item Spielberger State-trait Anxiety Inventory [37,38], where items were rated on a 4-point Likert scale (1 = not at all to 4 = very much) (e.g., “I feel calm”). Perceived stress was assessed using the 10-item Perceived Stress Scale [39,40], where items were rated on a 5-point Likert scale (0 = never to 4 = very often) (e.g., “In the last month, how often have you felt nervous or stressed?”). Detailed information about the two questionnaires can be seen in supplementary file for measures 2.4.2–2.4.3. Pre-tests and post-tests were conducted two days before and two days after the intervention project, respectively. Mid-tests were conducted on the weekends of the third intervention week. Cognitive functions were assessed in the pre- and post-tests with a valid cognitive test platform targeted at adults. The five computer-administered primary cognitive ability tests assessed included processing speed, working memory, episodic memory, visual-spatial ability, and a verbal ability test. The score for each cognitive task was normalized on a scale of 0–19 points based on results from over 100,000 subjects, with higher scores indicating better specific-domain cognitive function. Global cognitive function was taken as the sum of five cognitive tests, with scores ranging from 0 to 95. Participants in this study were asked to complete cognitive tasks in a quiet self-contained room between 2 and 5 p.m. It took them around 30 min to complete the five cognitive tasks. Details of the cognition measurements are described in supplementary file for measures 2.4.4–2.4.8.

### 2.5. Ethical Consideration

Ethical approval was obtained from the Institutional Review Board of Tsinghua University (ID number: 20190091). Detailed information about this study was presented clearly to every participant. Further, participants were informed that they could withdraw from this study at any time. All participants had read the study content and provided their digital informed consent before the intervention project began. Cognitive function assessments, based on the cognitive test platform, were offered to participants if they could complete this six-week project.

### 2.6. Statistical Analysis

All statistical analyses were performed using R software version 3.6.3 and IBM SPSS (Statistical Package for social science, Version 22, Chicago, IL, USA). First, for a baseline comparison between the intervention and waitlist groups, independent *t*-tests and chi-square tests were performed on continuous and categorical variables, respectively. Mixed-effect models with repeated measures were then implemented to analyze the effects of treatment (intervention vs. waitlist group); time (pre-test vs. mid-test and pre-test vs. post-test); and the interaction between treatment and time on behavioral, psychological, and cognitive dependent variables. The interaction effect indicated a changing effect of an intervention over time. We used a dummy variable to represent three timepoints and estimated its effect over time. For variables with significant between-group differences at baseline, we further compared the relative changes of two groups during the intervention period using independent *t*-tests. Finally, we estimated the effect of treatment at each timepoint with the Bonferroni correction. Partial Eta Squared ($\eta_{p}^{2}$) with values of 0.01, 0.06, and 0.14 were chosen as the thresholds to indicate small, medium, and large effect sizes, respectively [41,42]. A two-tailed *p*-value of <0.05 was considered statistically significant in all tests.

## 3. Results

### 3.1. Characteristics of the Survey Participants

This study included 62 young females with an average age of around 23 and over four years of high education. Most of the participants lived in urban areas (90.32%). No significant differences were observed in demographic, behavioral, psychological, and cognitive variables between the two groups at baseline, except for in the visual-spatial cognitive task, where the intervention group showed better visual-spatial ability (Table 1).

### 3.2. Effects of the Interventions on Health-Related Behaviors and Mental Health

Table 2 presents the results of intervention effectiveness on behavioral and psychological variables. For behavioral indices, significant main effects of time on physical activity (F = 22.36, *p* < 0.001, $\eta_{p}^{2}$ = 0.27), moderate-to-vigorous physical activity (F = 18.46, *p* < 0.001, $\eta_{p}^{2}$ = 0.24), and average sedentary time (F = 5.91, *p* = 0.004, $\eta_{p}^{2}$ = 0.09) were observed and the treatment × time interaction was shown to have a significant effect on moderate-to-vigorous physical activity (F = 4.20, *p* = 0.022, $\eta_{p}^{2}$ = 0.065). In addition, a significant increase in moderate-to-vigorous physical activity was observed in the intervention group from pre-test to post-test (M_diff_ = 940.61, *p* < 0.001, 95% confidence interval (95% CI): 576.67, 1304.55). In contrast, a significant main effect of treatment on the average sedentary time was shown (F = 4.69, *p* = 0.034, $\eta_{p}^{2}$ = 0.072) with the waitlist group reducing more than the intervention group at the time of the post-test (M_diff_ = −73.02, *p* = 0.038, 95% CI: −141.90, −4.14), which demonstrated that participants in the waitlist group made relatively more remarkable changes to their sedentary behaviors. Moreover, for psychological indices, significant main effects of time on anxiety (F = 7.73, *p* < 0.001, $\eta_{p}^{2}$ = 0.11) and perceived stress (F = 10.44, *p* < 0.001, $\eta_{p}^{2}$ = 0.15) were observed, but there were no significant treatment and interaction effects. Even so, greater changes in anxiety and perceived stress occurred in the intervention group during the six-week period (M_diff_ = −4.73, *p* = 0.002, 95% CI: −7.30, −2.15 and M_diff_ = −5.09, *p* = 0.001, 95% CI: −8.29, −1.89, respectively), which might imply a superiority of the intervention strategy combining HIIT with health education.

### 3.3. Changes of Cognitive Function Over the 6-Week Intervention

**Figure 3:**
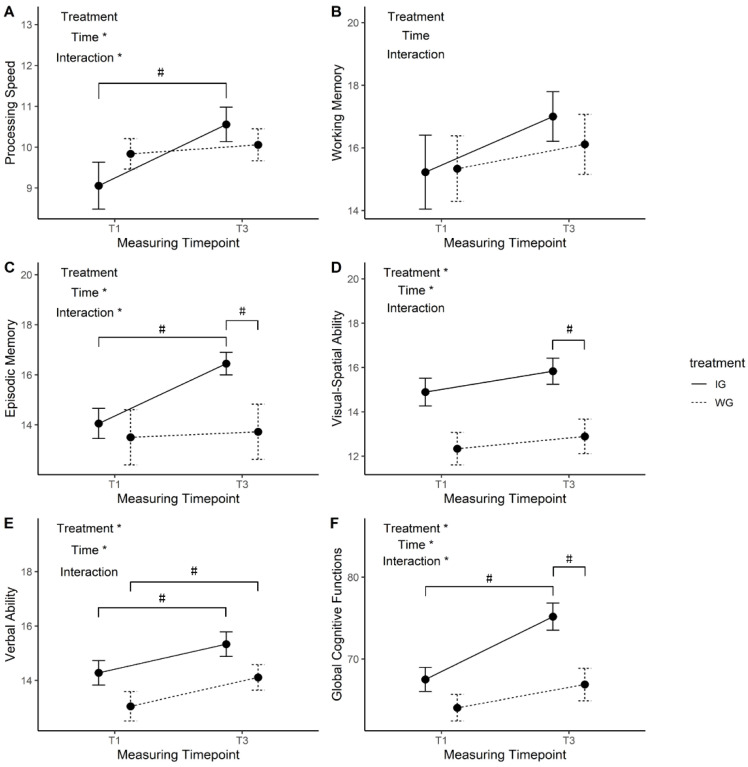
Effects of the treatment, time, and treatment x time interaction on cognitive function *^a,b^* Note: The subgroup of (**A**–**F**) indicates changes of two groups in processing speed, working memory, episodic memory, visual-spatial ability, verbal ability and global cognitive functions from pre-test to post-test. IG, intervention group, receiving HIIT and health education; WG, waitlist group, receiving health education only; T1, pre-test; T3, post-test. The score for each cognitive function test was converted into a norm of 0–19 points. The score for global cognitive function was the sum of five cognitive tasks, ranging from 0 to 95. * indicates a significant treatment, time and treatment × time interaction effect on a specific domain or global cognitive function. # represents a significant change from pre-test to post-test for each group or a significant difference between two groups in the post-test. ^a^ Mixed-effect models with repeated measures and analysis of variance *F* tests. ^b^ The degrees of freedom for between-participant, within-participant, and interaction-effect analyses were (1,34). The first degree of freedom in parentheses refers to the main or interactive effect and the second refers to the error term.

Figure 3 indicates the results of the intervention effectiveness on five primary cognitive functions. In this study, no significant treatment, time, or treatment × time effects on working memory were observed, whereas there were significant main effects of treatment on visual-spatial ability (F = 8.03, *p* = 0.008, $\eta_{p}^{2}$ = 0.19) and verbal ability (F = 8.03, *p* = 0.008, $\eta_{p}^{2}$ = 0.19). Although the treatment did not have significant main effects on episodic memory (F = 2.20, *p* = 0.15, $\eta_{p}^{2}$ = 0.061) or processing speed (F = 0.064, *p* = 0.80, $\eta_{p}^{2}$ = 0.002), there were significant treatment × time interaction effects on episodic memory (F = 4.21, *p* = 0.048, $\eta_{p}^{2}$ = 0.11) and processing speed (F = 4.11, *p* = 0.05, $\eta_{p}^{2}$ = 0.10). Furthermore, time was shown to have significant main effects on processing speed (F = 7.46, *p* = 0.010, $\eta_{p}^{2}$ = 0.18), episodic memory (F = 6.11, *p* = 0.019 $\eta_{p}^{2}$ = 0.15), visual-spatial ability (F = 4.21, *p* = 0.048, $\eta_{p}^{2}$ = 0.11), and verbal ability (F = 8.03, *p* = 0.008, $\eta_{p}^{2}$ = 0.19). Moreover, although the results of this intervention project showed different effects on various cognitive functions, in general, there were significant time (F = 21.24, *p* < 0.001, $\eta_{p}^{2}$ = 0.38), treatment (F = 7.85, *p* = 0.008, $\eta_{p}^{2}$ = 0.19), and treatment × time interaction (F = 4.50, *p* = 0.041, $\eta_{p}^{2}$ = 0.12) effects on global cognitive function. In addition, after the six-week period, participants in the intervention group showed significantly better episodic memory (M_diff_ = 2.72, *p* = 0.029, 95% CI: 0.30, 5.15), visual-spatial ability (M_diff_ = 2.94, *p* = 0.005, 95% CI: 0.96, 4.93), and global cognitive function (M_diff_ = 8.28, *p* = 0.003, 95% CI: 3.06, 13.50) in the post-test than those in the waitlist group. However, considering that the intervention group showed a significantly better visual-spatial ability at baseline than the waitlist group, we further compared the relative changes in the two groups. The results of independent *t*-tests showed that the HIIT intervention actually did not improve the visual-spatial ability of the IG better as there was no significant difference between groups (*t* = 0.14, *p* = 0.89).

## 4. Discussion

To our knowledge, this is the first study to investigate the effectiveness of a short-term online intervention on behavioral (i.e., PA, MVPA, and sedentary time), mental (i.e., perceived stress and anxiety), and cognitive (i.e., five specific and global cognitive functions, such as processing speed) health outcomes among young, sedentary Chinese women, and it might be the first to demonstrate that a “bodyweight HIIT + health education” intervention strategy is more effective for improving mental health and cognitive functions than the “health education alone” modality. The online characteristic of these intervention strategies has profound social implications. As we all know, the COVID-19 pandemic is sweeping the world, causing a global health burden [43]. Many people are suffering from increased sedentariness and negative emotions because of physical distancing [44]. Thus, the provision of a physical training intervention remotely via video conference makes this study highly relevant and important in current times, and it may benefit people from developing countries where offline physical training intervention is lacking but in dire need.

### 4.1. Effects of the Online Intervention on Health-Related Behaviors and Mental Health

We assumed that both the “bodyweight HIIT + health education” and “health education only” intervention modalities would increase health-related behaviors for sedentary young females, but we thought that the former would be a better strategy for improving mental health and cognitive function. This hypothesis was supported. Generally, both the intervention and waitlist groups demonstrated increased levels of physical activity and moderate-to-vigorous physical activity as well as decreased sedentary time. Participants in the intervention group, who underwent bodyweight HIIT-centered exercise, showed significant increases in participation in moderate-to-vigorous physical activity and significant reductions in negative emotions over time, while the waitlist group, who only received health education focusing on the risk of sedentariness, had significant decreases in sedentary time. Our results are consistent with previous offline and online physical training intervention studies where physical training interventions were shown to improve physical activity behaviors in sedentary females [18,21,22,45,46]. Moreover, our findings support a previous meta-analysis study that concluded that health interventions, such as health education, with a focus on reducing sedentary behaviors could lead to a meaningful reduction in sedentary time [47]. In addition, consistent with previous studies [48,49], we also found that physical training interventions, especially moderate-to-vigorous interventions, help to mitigate negative emotions in young adult women. Taken together, both an online bodyweight HIIT intervention combined with health education or health education alone can effectively induce health-related behaviors, although the exact health benefits may differ.

### 4.2. Changes in Domain-Specific and Global Cognition over the Six-Week Online Intervention

Moreover, both groups were found to have varying degrees of improved global cognitive function over the six-week intervention period, with the intervention group showing more remarkable changes. In detail, participants in the intervention group demonstrated more significant increases in processing speed and episodic memory and performed relatively better in the working memory test; although, these effects did not reach a significant level, probably because of the limited intervention period. Furthermore, the two groups showed similar uptrends in verbal ability and visual-spatial ability; although, these were not significant changes. As mentioned above, both groups demonstrated increased levels of PA over the 6-week intervention program. This finding is consistent with many review and intervention studies, showing that increased physical activity may contribute to improved cognitive function, and this has many possible underlying mechanisms, from molecular and cellular to brain morphological and behavioral changes [24,50,51,52,53,54]. For instance, experimental studies have found that exercise could exert its salutary effects on learning and memory by potentiating the cell activity of the hippocampal dentate gyrus, inducing neurotropic factors, such as BDNF and insulin-like growth factor-1 (IGF-1) to modulate hippocampal synaptic plasticity, thereby promoting the growth of hippocampal blood vessels and increasing the hippocampal volume [55,56,57]. The improvements in working memory, episodic memory, and visual-spatial ability measured in this study might be highly related to changes in the hippocampus. In addition, with the help of functional magnetic resonance imaging (fMRI), some randomized controlled trials (RCTs) have found that exercise interventions could improve executive functions (e.g., processing speed and working memory) and verbal ability by enhancing the functional connectivity of the prefrontal brain regions [58]. As to why the intervention group had a relatively greater increase in the cognitive ability tests, a limitation of the current study is that neurobiological processes leading to more pronounced increases in cognitive performance tests in the HIIT group were not assessed. In light of this limitation, further experiments investigating the influence of HIIT interventions on neurobiological processes that change cognitive performance are clearly needed [24]. However, some previous RCTs failed to identify cognitive benefits of physical activity. For example, in the LIFE randomized trial, a 24-month physical activity intervention involving walking and stretching was not found to be superior to a health education control in sedentary older adults [59]. Similarly, a six-month light-intensity PA intervention program provided a modest improvement in cognition among older adults with subjective cognitive impairment, but the positive effects disappeared over an 18-month follow-up period [47]. Possible explanations for these inconsistencies might include the following: (1) the intensity and modality of a physical activity intervention influences the changes in global or domain-specific cognitive function produced, although the intensity threshold is not clear [60,61,62]; (2) sensitivity to PA may vary among different groups, and young adults’ cognitive functions might be more sensitive to increased PA [59]; (3) PA interventions may only work in the short term, probably because the cortex might gradually adapt to PA over time, and the cognitive benefits of PA may attenuate and eventually vanish [59,63]. Despite this, in this study, it was interesting to discover that the online “bodyweight HIIT + health education” modality increased moderate-to-vigorous physical activity and improved cognitive functioning more effectively than the “health education only” intervention.

### 4.3. Limitations and Strengths

This study has several limitations. First, the sample size used in our randomized controlled trial was not very large. However, despite the use of this small data set, we still demonstrated the effectiveness of the bodyweight HIIT and health education intervention. Second, we did not explore the dose–response relationship between PA and cognitive improvement, and this deserves further investigation. Third, this study lacked cognitive biomarkers (e.g., BDNF and β-amyloid level) and brain images (e.g., fMRI) to further uncover underlying mechanisms. Fourth, due to the online characteristic, we failed to include objective measurements of PA and sedentary behavior (e.g., by accelerometer) and did not assess other physical parameters (e.g., body composition and muscular adaptation). Moreover, we used a subjective questionnaire measurement method, which might have resulted in a self-report bias. However, this type of structural questionnaire is widely used in many studies, so the results are generally considered to be reliable [33,38,39,64]. Furthermore, it was hard to monitor heart rate and control exercise intensity objectively in the online physical training courses, so we only subjectively judged participants’ exercise exertion with the help of the 0–10 RPE Borg Scale. This is an inevitable problem of remote interventions and RPE is quite commonly used. Lastly, this study was conducted during the COVID-19 pandemic in China. Subjects may have reported more negative emotions than usual. However, we do not think that this would have had any impact on the results, but it made this study more relevant and important. After all, the COVID-19 pandemic is still going on. Many people around the world may be suffering from physical and mental distress and may urgently need effective health interventions. In brief, despite some shortcomings, our online multicomponent intervention program provides a new perspective under which healthcare workers and professionals can develop health strategies. Moreover, this kind of low-cost intervention modality may soon spread out to and benefit more low and middle-income people with equal health requirements.

## 5. Conclusions

Among a group of sedentary young Chinese women, a six-week “bodyweight HIIT + health education” online intervention program resulted in a significant increase in moderate-to-vigorous physical activity and reduced negative emotions over time, while the “health education only” group, with greater emphasis on reducing sedentary behaviors, demonstrated a decrease in time spent doing sedentary behaviors. In addition, the intervention that combined bodyweight HIIT with health education resulted in more marked improvements in cognitive function. In a nutshell, these two short-term online intervention strategies can be useful to improve health-related behaviors, but the behavioral change consequences may differ. Moreover, in terms of improving mental health and cognitive function, an online bodyweight HIIT intervention combined with health education might be a relatively more effective modality than using health education alone.

## Figures and Tables

**Table 1:** Demographic characteristics and health outcomes of all participants at baseline.

Variables	Intervention Group (*n* = 33)	Waitlist Group (*n* = 29)	*p*
**Demographics**			
Age (years)	22.61 ± 2.19	22.83 ± 2.25	0.70
Body weight (kg)	58.13 ± 19.72	60.25 ± 19.55	0.67
Height (cm)	163.03 ± 7.13	165.67 ± 5.40	0.11
BMI (kg/m^2^)	21.79 ± 6.96	21.96 ± 7.10	0.93
Years of higher education	4.33 ± 1.67	4.34 ± 1.57	0.98
Region (city/countryside)	31/2	25/4	0.41
**Behavior**			
PA (METs-minutes/week)	693.89 ± 593.29	1011.53 ± 713.09	0.19
MVPA (METs-minutes/week)	318.79 ± 317.99	553.52 ± 853.17	0.16
Average ST (minutes/day)	369.16 ± 131.75	334.78 ± 146.13	0.30
**Psychology**			
STAI (score)	22.21 ± 6.80	20.97 ± 6.82	0.46
PSS (score)	19.15 ± 7.77	19.24 ± 6.72	0.96
**Cognition**			
Processing Speed (score)	9.06 ± 2.44	9.83 ± 1.58	0.21
Working Memory (score)	15.22 ± 1.12	15.33 ± 1.12	0.89
Episodic Memory (score)	14.06 ± 2.55	13.50 ± 4.68	0.72
Visual-spatial Ability (score)	14.89 ± 2.65	12.33 ± 3.11	0.01 *
Verbal Ability (score)	14.28 ± 1.90	13.06 ± 2.88	0.09
Global Cognitive Function (score)	67.50 ± 6.23	64.06 ± 6.82	0.20

Note: BMI, body mass index, calculated by the formula 10,000 × weight (kg)/height (cm)^2^. PA, physical activity (metabolic equivalents (METs)-minutes/week); MVPA, moderate-to-vigorous physical activity (METs-minutes/week); Average ST, average sedentary time (minutes/day); STAI, Spielberger State-Trait Anxiety Inventory, ranging from 10 to 40 [31,32]; PSS, Perceived Stress Scale, ranging from 0 to 40 [33,34]. Continuous variables are expressed as the Mean ± Standard Deviation. * indicates a significant difference at *p* ≤ 0.05.

**Table 2:** Effects of treatment, time, and interaction on behavior and psychological variables ^a^.

Variables	Mean Difference (*p*)		*p* ($\eta_{p}^{2}$) for the Mixed-Effect Model ^b^		
	Intervention Group (*n* = 33)	Waitlist Group (*n* = 29)	Between- Participants	Within- Participants	Interaction- Effect
PA					
ΔPA_T2-T1_	994.70 (<0.001) *	690.40 (0.010) *	0.73 (0.002)	<0.001 * (0.27)	0.31 (0.019)
ΔPA_T3-T1_	1218.00 (<0.001) *	738.97 (0.003) *			
T2_IG-WG_	−13.36 (0.96)				
T3_IG-WG_	161.40 (0.51)				
MVPA					
ΔMVPA_T2-T1_	718.79 (<0.001) *	221.66 (0.69)	0.22 (0.024)	<0.001 * (0.24)	0.022 * (0.065)
ΔMVPA_T3-T1_	940.61 (<0.001) *	358.90 (0.08)			
T2_IG-WG_	282.40 (0.13)				
T3_IG-WG_	346.98 (0.072)				
AST					
ΔAST_T2-T1_	−66.95 (0.05) *	−73.99 (0.05) *	0.034 * (0.072)	0.004 * (0.090)	0.61 (0.008)
ΔAST_T3-T1_	−24.92 (0.95)	−63.56 (0.13)			
T2_IG-WG_	41.42 (0.16)				
T3_IG-WG_	73.02 (0.038) *				
STAI (score)					
ΔSTAI_T2-T1_	−4.03 (0.027) *	−1.45 (0.94)	0.81 (0.001)	<0.001 * (0.11)	0.38 (0.016)
ΔSTAI_T3-T1_	−4.73 (0.002) *	−2.69 (0.18)			
T2_IG-WG_	−1.34 (0.40)				
T3_IG-WG_	−0.79 (0.64)				
PSS (score)					
ΔPSS_T2-T1_	−2.39 (0.35)	−1.89 (0.73)	0.69 (0.003)	<0.001 * (0.15)	0.89 (0.002)
ΔPSS_T3-T1_	−5.09 (0.001) *	−4.14 (0.02) *			
T2_IG-WG_	−0.59 (0.77)				
T3_IG-WG_	−1.04 (0.54)				

Note: IG, intervention group; WG, waitlist group; PA, physical activity (METs-minutes/week); MVPA, moderate–vigorous physical activity (METs-minutes/week); AST, average sedentary time (minutes/day); STAI, Spielberger State-Trait Anxiety Inventory, ranging from 10 to 40; PSS, Perceived Stress Scale, ranging from 0 to 40; T1, pre-test; T2, mid-test; T3, post-test. Each variable included four comparison analysis results. For example, ΔPA_T2-T1_ and ΔPA_T3-T1_ indicate within-group changes in PA from pre-test to mid-test and from pre-test to post-test for each group, while T2_IG-WG_ and T3_IG-WG_ represent between-group differences in PA at mid-test and post-test. * indicates a significance at *p* ≤ 0.05. ^a^ Mixed-effect model with repeated measures and analysis of variance *F* tests. ^b^ The degrees of freedom for between-participant, within-participant, and interaction-effect analyses were (1,60), (2,120), and (2,120), respectively. The first degree of freedom in parentheses refers to the effect (between different groups or between different measuring times or interactions) and the second refers to the error term.

## Data Availability

The data that support the findings of this study are available from the corresponding author, upon reasonable request.

## Supplementary Materials

The following are available online at https://www.mdpi.com/1660-4601/18/1/302/s1, Table S1: Intervention Plans for HIIT Courses and Health Education for Two Groups during a 6 Week intervention period. Supplementary file: Measures.
